# Visualization of root extracellular traps in an ectomycorrhizal woody plant (*Pinus densiflora*) and their interactions with root-associated bacteria

**DOI:** 10.1007/s00425-023-04274-1

**Published:** 2023-11-07

**Authors:** Makoto Shirakawa, Norihisa Matsushita, Kenji Fukuda

**Affiliations:** 1 Graduate School of Agricultural and Life Sciences, The University of Tokyo, 1-1-1 Yayoi, Bunkyo-ku, Tokyo, 113-8657 Japan; 2 Japan Society for the Promotion of Science, 5-3-1 Kojimachi, Chiyoda-ku, Tokyo, 102-0083 Japan

**Keywords:** Border cells, Japanese red pine, *Paraburkholderia*, Primary root, Rhizobacteria, Root mucilage

## Abstract

**Main conclusion:**

Extracellular traps in the primary root of *Pinus densiflora* contribute to root-associated bacterial colonization. Trapped rhizobacteria induce the production of reactive oxygen species in root-associated, cap-derived cells.

**Abstract:**

Ectomycorrhizal (ECM) woody plants, such as members of Pinaceae and Fagaceae, can acquire resistance to biotic and abiotic stresses through the formation of mycorrhiza with ECM fungi. However, germinated tree seedlings do not have mycorrhizae and it takes several weeks for ectomycorrhizae to form on their root tips. Therefore, to confer protection during the early growth stage, bare primary roots require defense mechanisms other than mycorrhization. Here, we attempted to visualize root extracellular traps (RETs), an innate root defense mechanism, in the primary root of *Pinus densiflora* and investigate the interactions with root-associated bacteria isolated from ECM and fine non-mycorrhizal roots. Histological and histochemical imaging and colony-forming unit assays demonstrated that RETs in *P*. *densiflora*, mainly consisting of root-associated, cap-derived cells (AC-DCs) and large amounts of root mucilage, promote bacterial colonization in the rhizosphere, despite also having bactericidal activity via extracellular DNA. Four rhizobacterial strains retarded the mycelial growth of a pathogenic strain belonging to the *Fusarium oxysporum* species complex in dual culture assay. They also induced the production of reactive oxygen species (ROS) from host tree AC-DCs without being excluded from the rhizosphere of *P*. *densiflora*. Applying three *Paraburkholderia* strains, especially PM O-EM8 and PF T-NM22, showed significant differences in the ROS levels from the control group. These results reveal the indirect contributions of rhizobacteria to host root defense and suggest that root-associated bacteria could be a component of RETs as a first line of defense against root pathogens in the early growth stage of ECM woody plants.

**Supplementary Information:**

The online version contains supplementary material available at 10.1007/s00425-023-04274-1.

## Introduction

Plants have root apical meristems (RAMs) on the root tip, which is a vital organ that controls cellular division and differentiation (Motte et al. 2019). Roots are constantly exposed to various biotic and abiotic stresses during elongation through soil (Cramer et al. 2011; Suzuki et al. 2014; Ganesh et al. 2022). Among stressors, pathogen attack is a major cause of mortality of juvenile plants (Atkinson and Urwin 2012; Gonthier and Nicolotti 2013). Plants repel or mitigate pathogen invasion and infection via innate defense responses, such as pathogen/microbe-associated molecular pattern (PAMP/MAMP)-triggered immunity (Huot et al. 2014; Cook et al. 2015). Furthermore, numerous studies have shown that host roots recruit nonpathogenic, root-associated bacteria by releasing various root exudates (Badri and Vivanco 2009; Sasse et al. 2018). Such bacteria produce exopolysaccharides (EPSs), antipathogenic metabolites, and phytohormones, which in turn help control the rhizosphere environment (De Vleesschauwer and Höfte 2009; Kuzyakov and Razavi 2019; Mohanram and Kumar 2019). With the development of high-throughput sequencing technologies, we have come to better understand the interactions between host plants and pathogenic, commensal, and mutualistic rhizospheric microorganisms, as well as their impacts on the health and productivity of host plants.

Rhizodeposits include water-soluble and volatile compounds that are released from host roots, and are involved in biological defense. The root cap is made up of root border cells (RBCs) and root border-like cells (RBLCs), which cover vital tissues and release single or connected cells into the soil. Collectively, RBCs and RBLCs are termed root-associated, cap-derived cells (AC-DCs) (Hawes and Lin 1990; Hawes et al. 2003; Driouich et al. 2019). Studies have revealed that these cells remain alive after release from the root cap (Hawse and Pueppke 1986) and secrete mucilage composed of a mixture of polysaccharides, proteoglycans, extracellular DNA (exDNA), defensin peptides, reactive oxygen species (ROS), and various secondary metabolites (Plancot et al. 2013; Wen et al. 2017; Driouich et al. 2019, 2021). These structures, consisting of AC-DCs and their secretions that spread to encompass the root cap, are known as root extracellular traps (RETs) because of similarities with neutrophil extracellular traps, which are part of the innate immune system in animals (Brinkmann et al. 2004; Driouich et al. 2013). Studies on RETs have mainly focused on their defense functions against root pathogens. Most studies have used herbaceous plant species, such as *Arabidopsis* (*Arabidopsis thaliana* [L.] Heynh.), cotton (*Gossypium* spp.), maize (*Zea mays* L.), and pea (*Pisum sativum* L.) (Hawes et al. 2003, 2016; Vicré et al. 2005; Wen et al. 2009; Fortier et al. 2023). By contrast, the roles of RETs in woody plants remain poorly understood, and only a few cases have been investigated, including in *Acacia mangium* Willd. (Endo et al. 2011), grapevine (*Vitis riparia* × *Vitis labrusca*) (Liu et al. 2019), and some species in arid regions (*Balanites aegyptiaca* [L.] Del., *Acacia raddiana* Savi, and *Tamarindus indica* L.) (Carreras et al. 2020).

Pinaceae is a representative ectomycorrhizal (ECM) tree family that inhabits temperate and boreal forests in the Northern Hemisphere. This family acquires resistance to biotic and abiotic stresses, including pathogen attacks, via below ground ECM associations. Such resistance allows Pinaceae species to invade non-forested sites and survive in the seedling stage (Martín-Pinto et al. 2006; Zhang et al. 2017; Policelli et al. 2019). However, ECM fungi generally associate with only fine root tips (≤ 2 mm in diameter), which are generated from lateral roots. This suggests that there is a period between seed germination and ECM root formation of weakened defense responses during root formation. Therefore, we hypothesized that other defense mechanisms confer protection in the early growth stage, and focused our investigations on RETs and root-associated bacteria.

We elucidated the features and roles of RETs in the early stage of germination of ECM tree species. To this end, we visualized the RETs of an ECM woody gymnosperm, Japanese red pine (*Pinus densiflora* Sieb. et Zucc.), investigated whether RETs in the host tree species kill root-associated bacteria or promote their colonization during early growth, and evaluated the effects of those bacteria on the defense responses of host tree AC-DCs.

## Materials and methods

### Plant material

**Fig. 1:**
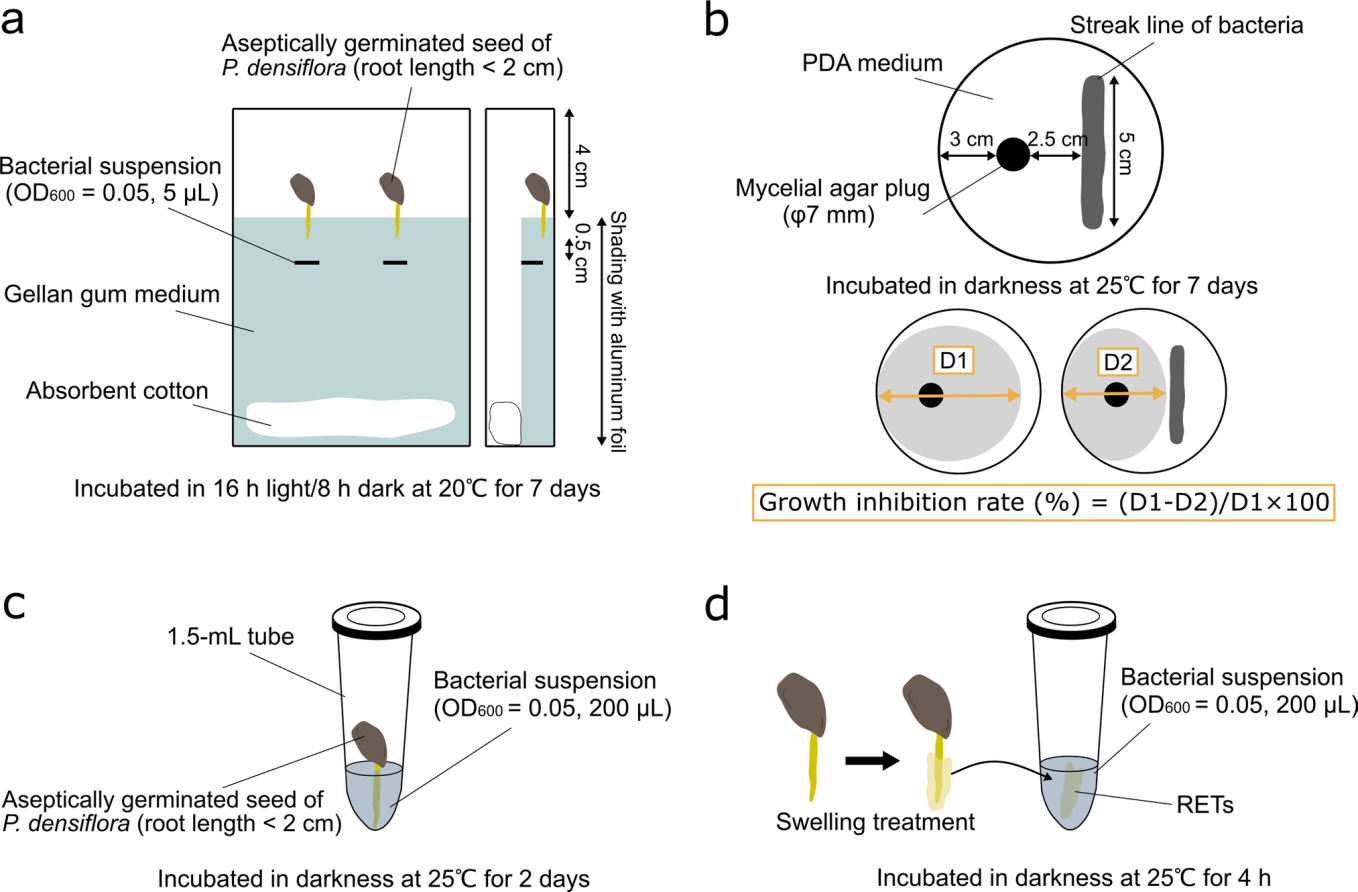
Schematic overview of the experimental design used in this study. **a** Inoculation assay to evaluate the effects of the selected rhizobacterial strains on the root and shoot growth of *Pinus densiflora*. **b** Dual culture assay of the bacterial strains against plant pathogenic fungus *Fusarium* sp. F-1 (the *Fusarium oxysporum* species complex, MAFF No. 235711). **c** Co-culture system of the aseptically germinated seed of *P. densiflora* and the bacterial strains for microscopic observations and colony-forming unit assay. **d** Co-culture system of root-associated, cap-derived cells (AC-DCs) and bacterial strains for the detection of reactive oxygen species (ROS) from AC-DCs in response to contact with them

**Fig. 2:**
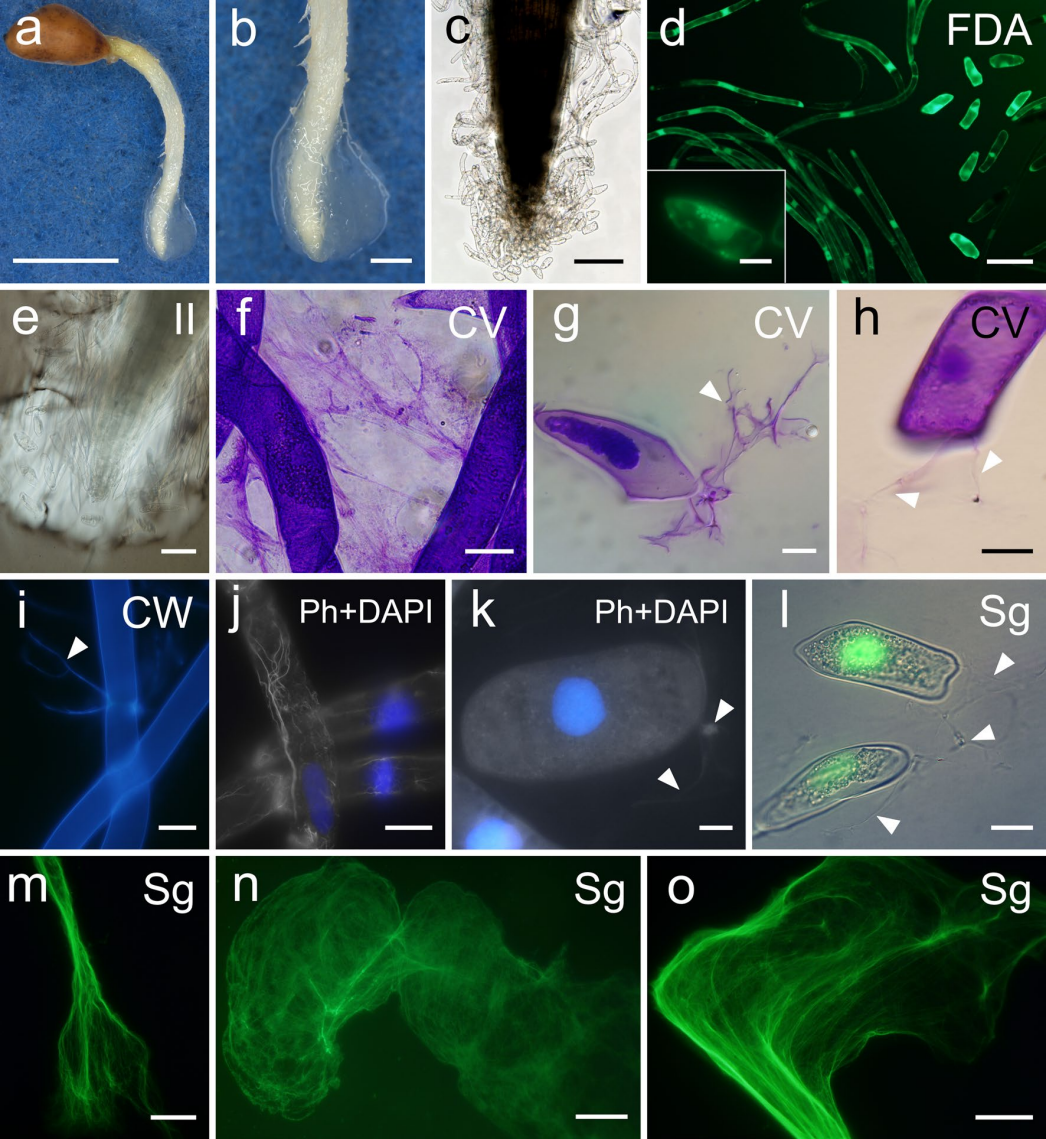
Histological and histochemical imaging of the main components of the root extracellular traps (RETs) in the early growth stage of *Pinus densiflora*. **a**, **b** A root tip after swelling treatment. **c** A root cap and AC-DCs. **d** Cell viability of AC-DCs after detachment from the root cap. **e**, **f** Root mucilage secretions stained with India ink (**e**) and crystal violet (**f**) solutions. **g**–**i** Branched strands protruding from dead (**g**) and living (**h**, **i**) cells. **j**, **k** Merged images of actin filaments (gray) and nuclei (blue) in AC-DCs. **l** Nuclei of dead cells and strands protruding from cells. **m**–**o** Extracellular DNA spread in a thread-like or web-like structure. The abbreviations in the upper right corner of each image indicate the staining solutions applied: FDA, fluorescein diacetate solution; II, India ink; CV, crystal violet; CW, calcofluor white; Ph, Acti-stain 555 fluorescent phalloidin; DAPI, 4′,6-diamidino-2-phenylindole; and Sg, SYTOX Green. White arrowheads point to branched strands. The images in **c** and **l** were adjusted for brightness and contrast using GIMP. Bars = 5 mm (**a**), 1 mm (**b**), 200 µm (**c**), 100 µm (**d** [lower right], **e**), 50 µm (**n**), 20 µm (**d** [lower left], **f**, **h**–**j**, **l**, **m**, **o**), 10 µm (**g**, **k**)

*Pinus densiflora* seeds were immersed in distilled water in darkness at 4 ℃ overnight (for 12–18 h). They were shaken in a neutral detergent solution for 1 min using a magnetic stirrer and rinsed with tap water. Intact seeds were surface sterilized with 30% (v/v) hydrogen peroxide for 30 min and rinsed several times with sterile water; then they were sown on sterile filter paper moistened with sterile water and incubated under aseptic conditions in darkness at 25 ℃ for 4–14 days (mainly those germinated on the fourth or fifth day of culture with a root length < 2 cm were used). Before the separation of RETs from the root cap for microscopic observation or experimentation, germinated seeds were soaked on sterile filter paper with sterile water and incubated overnight under the same conditions as described above. Hereafter, this process is referred to as the swelling treatment (Figs. 1d, 2a, b).

### Histochemical staining and microscopy of RETs

Root tips from *P*. *densiflora* in the early germination stage were mounted on a glass slide for stereomicroscopy and light microscopy. Components of RETs were mounted in sterile water or following staining on a glass slide for bright-field and fluorescence microscopy using a light microscope (Olympus BX50; Olympus, Tokyo, Japan). All staining and observation processes were conducted on at least three technical replicates.

To visualize the viability of AC-DCs shed from the root cap, live and dead cells were stained with fluorescein diacetate solution (Dojindo Laboratories, Kumamoto, Japan; 1 µg mL^−1^ in phosphate-buffered saline [PBS]) and 0.01% (v/v) Evans blue (Wako Pure Chemical Ind., Ltd., Osaka, Japan) solution, respectively. Cells were stained and observed within 15 min of separation from the root. The same process was also performed on the separated samples incubated in a 1.5-mL tube containing 200 µL sterile water at 25 ℃ for 2, 4, 7, 14, and 28 days. Five replicates were prepared for each time point.

AC-DCs were stained using 1% or 3% (v/v) crystal violet (CV) solution (Hayashi Pure Chemical Ind., Ltd., Osaka, Japan), and the mucilage layer was visualized using CV of the same concentration and 0.4% (v/v) India ink solution; sterile water was used as the solvent for both solutions.

The CV solution and calcofluor white M2R (Sigma Chemical Co., St. Louis, MO, USA) solution (1 mg mL^−1^ in sterile water) were used to clarify the origin of branched strands frequently found as AC-DCs shed. In addition to these two solutions, we used Acti-stain 555 fluorescent phalloidin (Cytoskeleton Inc., Denver, CO, USA) under the following conditions at room temperature to visualize the same target. Specifically, cells were fixed with 4% (v/v) paraformaldehyde in PBS (pH 7.0) for 15 min, rinsed with PBS for 30 s, incubated in 0.1% (v/v) Triton X-100 solution (Nacalai Tesque Inc., Kyoto, Japan) in PBS for 15 min, rinsed with PBS for 30 s twice, stained with 0.1 µM phalloidin for 30 min, rinsed with PBS for 30 s three times, stained with 4′,6-diamidino-2-phenylindole (DAPI) as a counterstain for 30 s, and rinsed with PBS for 30 s.

ExDNA released from AC-DCs was labeled using 1 µM SYTOX Green (Thermo Fisher Scientific, Waltham, MA, USA). Stock solutions were made following the manufacturer’s instructions.

### Isolation of rhizobacteria from the root tips of *P. densiflora*

To obtain rhizobacteria, we collected root system samples of mature *P*. *densiflora* trees in August 2020 at Ome Forest, a temperate secondary forest in Ome, Tokyo, Japan (35°47′50.4″N, 139°15′44.4″E), and the University of Tokyo Tanashi Forest, a planted forest in Nishitokyo, Tokyo, Japan (35°44′21.0″N, 139°32′15.0″E). The samples were gently washed in tap water with a brush under a stereomicroscope (Leica MZ16; Leica Microsystems, Wetzlar, Germany). Five ECM root tips of the same morphotypes and the same number of non-mycorrhizal (NM) root tips (approximately 2–5 mm in length) were selected from each sample. They were transferred to a 1.5-mL tube containing 1 mL sterile water and shaken for 1 min using a vortex to remove fine particles. After repeating this process three times, each root tip was homogenized using a micropestle and suspended in 1 mL sterile water. The suspension was used as a stock solution, which was serially diluted up to 10,000 times, and 100 µL of each dilution was spread on a yeast glucose (YG) agar medium containing 1.0 g yeast extract, 1.0 g glucose, 0.3 g K_2_HPO_4_, 0.3 g KH_2_PO_4_, 0.2 g MgSO_4_·7H_2_O, 15 g agar, and 1 L distilled water (pH 7.0). All medium plates were incubated in darkness at 25 ℃ for 2–7 days, and bacterial colonies generated on the plate were randomly isolated.

### Selection and molecular identification of rhizobacterial strains

According to Shirakawa et al. (2019), of the culturable bacteria isolated from the root tips of *P. densiflora* with our method, mucoid-type colonies and non-mucoid (mousse)-type ones are easily distinguished as genus *Paraburkholderia* and *Bacillus*, respectively: the former appears predominantly on the medium, while the other is less dominant but frequently observed in each sample. Both genera are known to plant growth-promoting rhizobacteria (Santoyo et al. 2016) and mycorrhiza helper bacteria (Frey-Klett et al. 2007), and have been reported to promote seed germination and early growth in *Pinus* species (Domínguez-Castillo et al. 2021). In particular, *Paraburkholderia* is one of the dominant genera in the roots of *Pinus* (Nguyen and Bruns 2015) and has been suggested to have beneficial properties for host plants, including the production of indole-3-acetic acid and antifungal compounds (Compant et al. 2008; Ravi et al. 2021). Based on these findings, we selected three *Paraburkholderia* strains and one *Bacillus* strain in this study.

Molecular identification of the four bacterial strains was based on the 16S ribosomal RNA (rRNA) gene sequence. The V1–V9 regions of the 16S rRNA genes were amplified by direct polymerase chain reaction (PCR) from a single colony using EmeraldAmp PCR Master Mix (Takara Bio, Shiga, Japan) and the universal primer pair 27F and 1492R (Weisburg et al. 1991). The PCR cycling conditions were as follows: denaturing at 94 ℃ for 1 min; 40 cycles of 98 ℃ for 10 s, annealing at 55 ℃ for 30 s, and 72 ℃ for 90 s, and a final extension at 72 ℃ for 7 min. Successfully amplified PCR products were purified using Illustra ExoProStar (GE Healthcare, Buckinghamshire, UK) and submitted to Macrogen DNA Sequencing Service (Macrogen, Tokyo, Japan) for Sanger sequencing. Four universal primers (27F, 518F, 800R, and 1492R) were used as sequencing primers to obtain nearly complete sequences (> 1300 bp). After checking the quality of the obtained sequences with reference to the original chromatograms, bacterial species were identified at least to the genus level via a BLAST search of the GenBank database (http://blast.ncbi.nlm.nih.gov/Blast.cgi). These sequences were deposited in the DNA Database of Japan (https://www.ddbj.nig.ac.jp/index-e.html) under the accession numbers LC743733–LC743736. Table 1 lists the four bacterial strains used in this study. They were pre-cultured on YG agar medium in darkness at 25 ℃ for 2 days before use in each assay.

**Table 1:** Root-associated bacterial strains isolated from the root tips of *Pinus densiflora*

Strain	Abbreviation	Isolation source (location)	Accession no.	Top BLAST hit in GenBank (acc. no.)	Similarity (%)
*Bacillus* sp. strain O-EM7	BC O-EM7	ECM root tips (Ome, Tokyo)	LC743733	*Bacillus cereus* strain CASMBAUDAL1 (KM524118)	99.58
*Paraburkholderia* sp. strain O-EM8	PM O-EM8	ECM root tips (Ome, Tokyo)	LC743734	*Paraburkholderia metrosideri* strain 17G39-22 (MH934925)	100
*Paraburkholderia* sp. strain O-NM9	PS O-NM9	NM root tips (Ome, Tokyo)	LC743735	*Paraburkholderia sediminicola* strain HU2-65W (MN727305)	99.18
*Paraburkholderia* sp. strain T-NM22	PF T-NM22	NM root tips (Tanashi, Tokyo)	LC743736	*Paraburkholderia* sp. JSA6 (LC682224)	99.66

*ECM* ectomycorrhizal, *NM* non-mycorrhizal

### Inoculation of rhizobacterial strains on host tree root

Four rhizobacterial strains were inoculated on the early growth stage of *P. densiflora* to determine their effects on host tree growth and health. They were pre-cultured as mentioned above, then suspended in sterile water and adjusted to an optical density at 600 nm (OD_600_) of 0.05. Two aseptically germinated seeds were transplanted in square Petri dishes (144 × 100 × 16 mm^3^) with gellan gum medium containing 0.1 g yeast extract, 0.03 g K_2_HPO_4_, 0.03 g KH_2_PO_4_, 1.0 g MgSO_4_·7H_2_O, 2.8 g gellan gum, and 1 L distilled water (pH 5.5–6.5). Incisions were made in the medium just below the tips of both roots with a sterile scalpel, and 5 µL bacterial suspension was inoculated (Fig. 1a). Sterile water was used as the control, and five replicates of each treatment were prepared. Each plate was covered with aluminum foil, the part containing the medium, and grown in a growth chamber under 16 h light/8 h dark at 20 ℃. At 7 days after inoculation, the root and shoot lengths were measured and checked to determine whether there were any symptoms, such as wilting, browning, or root rot. The growth increment (cm) of the root and shoot were calculated based on the following formula: {(LA1−LT1) + (LA2−LT2)}/2, where LA is the root or shoot length after culturing, and LT is the root or shoot length at transplant.

### Dual culture assay

The anti-pathogenic activity of selected bacterial strains was tested by dual culture assay. We obtained *Fusarium* sp. F-1 (the *Fusarium oxysporum* species complex, MAFF No. 235711) from the NARO Genebank, Japan, as the plant pathogenic fungus used for the assay. This fungal strain was pre-cultured on a low carbon agar (LCA) medium (Miura and Kudo 1970) containing 0.2 g yeast extract, 1.0 g glucose, 2.0 g NaNO_3_, 1.0 g KH_2_PO_4_, 0.2 g KCl, 0.2 g MgSO_4_·7H_2_O, 18 g (originally 13 g) agar, and 1 L distilled water (pH 6.5–7.0), in darkness at 25 ℃ for 10 days. A mycelial agar plug (7 mm in diameter) of the pathogen was placed on one side of a potato dextrose agar medium (PDA; Eiken Chemical Co. Ltd., Tokyo, Japan) plate (90 mm in diameter), and pre-cultured bacterial colonies were streaked on the other using a 10-µL sterile disposable loop (Fig. 1b). Only the mycelium plug placed served as the control. Five replicates per bacterial strain were incubated in darkness at 25℃ for 7 days. The growth inhibition rate of each bacterial strain was calculated based on the following formula: growth inhibition rate (%) = (D1−D2)/D1 × 100, where D1 is the diameter of mycelium on the control, and D2 is the diameter of mycelium co-cultured with the bacterial strains.

### Visualization of rhizobacteria trapped by RETs

For the observations, four bacterial suspensions (OD_600_ = 0.05), mainly *Bacillus* sp. strain O-EM7 (BC O-EM7) and *Paraburkholderia* sp. strain O-NM9 (PS O-NM9), were prepared as mentioned above. Next, the tip of a primary root was immersed in a 1.5-mL tube containing 200 µL suspension and was incubated in darkness at 25 ℃ for 2 days (Fig. 1c). After incubation, RETs were detached from the root cap using tweezers, rinsed with sterile water twice, and then stained with 3% CV solution and 1 µM SYTOX Green.

### Colony-forming unit assay

We performed a colony-forming unit (CFU) assay to confirm the effects of the presence or absence of RETs on bacterial colonization in the early stage of *P*. *densiflora* rhizosphere development. Root tips, processed via swelling treatment, were immersed in bacterial suspensions (OD_600_ = 0.05), and RETs were removed under aseptic conditions before and 2 days after incubation in darkness at 25 ℃, respectively (Fig. 1c). Root tips with RETs were used as the control (*n* = 5). Incubated samples were separated from the seed, leaving a root tip of 5 mm, gently rinsed with sterile water twice, homogenized using a micropestle, and suspended in 1 mL sterile water. The suspensions were used as a stock solution, serially diluted up to 100,000 times, and 100 µL each dilution was spread on two dishes of YG agar medium per dilution step. After incubation at 25 ℃ for 2 days, bacterial colonies formed on the medium were counted; the average number of colonies in two dishes that fell within 30–300 was adopted, and the values were log-transformed (Log10 CFU) for statistical analysis.

### Detection of total ROS in response to contact with rhizobacteria

To evaluate the defense response of the RETs induced by contact with rhizobacteria, we examined ROS production, which is an early defense signal in plant root immunity (Boller and Felix 2009). After swelling treatment, RETs were stripped from the root tip, immersed in 200 µL each bacterial suspension (OD_600_ = 0.05), and incubated in darkness at 25 ℃ for 4 h (Fig. 1d). In addition, RETs were treated with 1 µM flg22 (Alpha Diagnostic International Inc., San Antonio, TX, USA), a representative MAMP peptide derived from bacterial flagella (Millet et al. 2010). Sterile water was used as the control, and five replicates of each treatment were performed. After rinsing twice with Hank’s balanced salt solution without phenol red (HBSS [−]), RETs were stained using the fluorescent probe ROS Assay Kit -Highly Sensitive DCFH-DA- (Dojindo Laboratories) at 25 ℃ for 30 min. The working solution was prepared following the manufacturer’s instructions. Stained samples were rinsed twice with HBSS-, and then five random locations per sample were imaged under fluorescence microscopy within 2 h of staining using the following conditions (except when images were captured at 1000 × magnification): 2040 × 1536 pixels; 200 × magnification; ISO200; 12 ms exposure; RGB values of 0.7:1.0:2.1; 8 bits; and .tiff extension. Total ROS production, here considered to indicate the degree of cellular sensitivity to the treatments, was calculated as the relative fluorescence intensity per sample, using Fiji (ImageJ ver. 1.53q) software (Schindelin et al. 2012). First, the original image was divided into three colors (red, blue, and green), and red was subtracted from green. Then, an image was generated using the Li model and a range of 25–255 as the threshold value. Finally, the number of pixels corresponding to the threshold was counted.

### Image processing

Fiji, Inkscape ver. 1.0 (https://inkscape.org/), and GIMP ver. 2.10.20 (https://www.gimp.org/) software were used to export and process each figure. Except for those used for the image analyses, when processing images after capturing, the entire image was altered and only brightness and contrast were modulated. Movies (Online Resources S1–S8) were processed using DaVinci Resolve ver. 18.1.2 (https://www.blackmagicdesign.com/products/davinciresolve/) under the same conditions.

### Statistical analysis

The significance of differences among treatments in each assay was analyzed by a one-way analysis of variance followed by a Tukey’s honest significant difference (HSD) test. Data processing and analysis and graph plotting were performed using R ver. 4.3.0 (R Core Team 2023).

## Results

### Visualization of RETs in the primary root of *P*.* densiflora*

Figure 2 presents an overview of the RETs and their major components in the primary root of *P*. *densiflora*. RBC shedding, RBLC detachment, and root mucilage percolation from the root cap were immediately observed when root tips that had not yet undergone swelling treatment were immersed in the solutions (Fig. 2c, e, and f; Online Resources S1 and S2). The root cap released mainly elliptic and oblong RBCs from the apex, and sheath-shaped and long layers of RBLCs from the lateral sides. CV readily stained each component, revealing their dispersal to cover the root tip; membranous mucilage was visualized particularly well (Figs. 2f, 5). Most of the AC-DCs remained viable in sterile water immediately after isolation from the root cap (Fig. 2d). We confirmed vigorous cytoplasmic streaming initially and even after 7 days of isolation (Online Resources S3–S5); some cells remained viable until day 28 (Fig. S1). However, cells mounted without liquid wilted and died quickly. Branched strands were observed to protrude from both living and dead cells, particularly at the joints between RBLCs and at the longitudinal tip of the RBCs (Fig. 2g–i). Damaged AC-DCs experiencing plasmolysis tended to discharge copious amounts of strands compared to live cells (Fig. 2g). They were readily stained with CV and calcofluor white (CW), and slightly stained with Acti-stain 555 phalloidin (Fig. 2g–i, k). Actin filaments in the AC-DCs had structures similar to the strands, but we could not confirm whether they were identical (Fig. 2j, k). Additionally, no labeling was observed after applying two fluorescent dyes for DNA, DAPI and SYTOX Green (Fig. 2j–l). SYTOX Green stained exDNA, which visualized their spread in thread-like or web-like structures (Figs. 2m–o, 6). These structures were observed during unraveling of the spherical structure of the cell nucleus (Fig. 6a), and some structures spread more than five times the area of a single RBC (Fig. 6d). The unfolding of exDNA was found both with and without microorganisms, and we could not confirm active secretion during our observations.

### Evaluation of the properties of rhizobacterial strains for the host tree growth and health

**Fig. 3:**
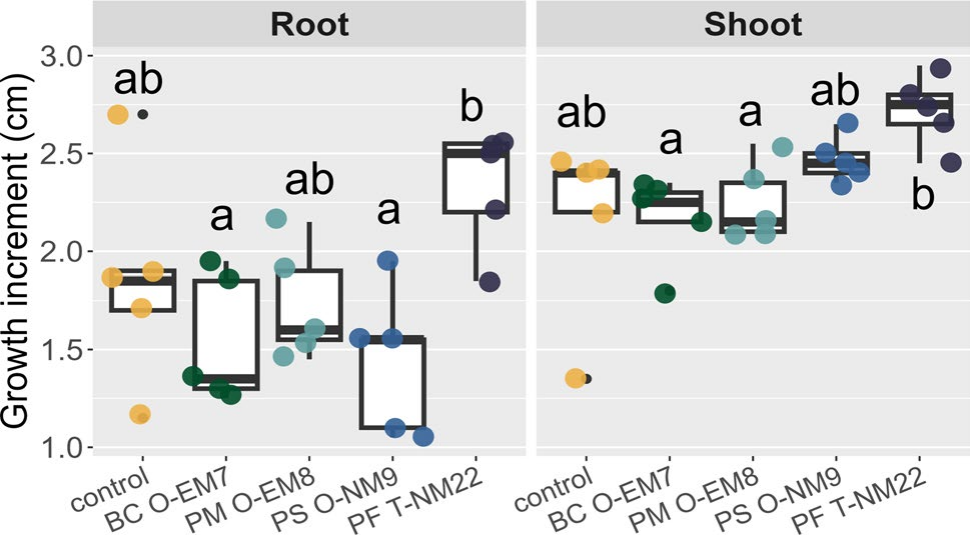
Root and shoot growth increments of *Pinus densiflora* after 7 days of inoculation of the rhizobacterial strains (*n* = 5) *Bacillus* sp. strain O-EM7 (BC O-EM7), *Paraburkholderia* sp. strain O-EM8 (PM O-EM8), *Paraburkholderia* sp. strain O-NM9 (PS O-NM9), and *Paraburkholderia* sp. strain T-NM22 (PF T-NM22). Different letters (a, b) indicate significant (*P* < 0.05) differences among the treatments according to Tukey’s HSD test

The effects of inoculating rhizobacteria on the root and shoot growth of *P. densiflora* are shown in Fig. 3. No significant differences were found in either inoculation treatment compared to the control at 7 days of cultivation. Among four bacterial strains, PF T-NM22 differed significantly from BC O-EM7 and PS O-NM9 in root length and BC O-EM7 and PM O-EM8 in shoot length. Moreover, none of the symptoms were observed by inoculation in any of the treatments.

**Fig. 4:**
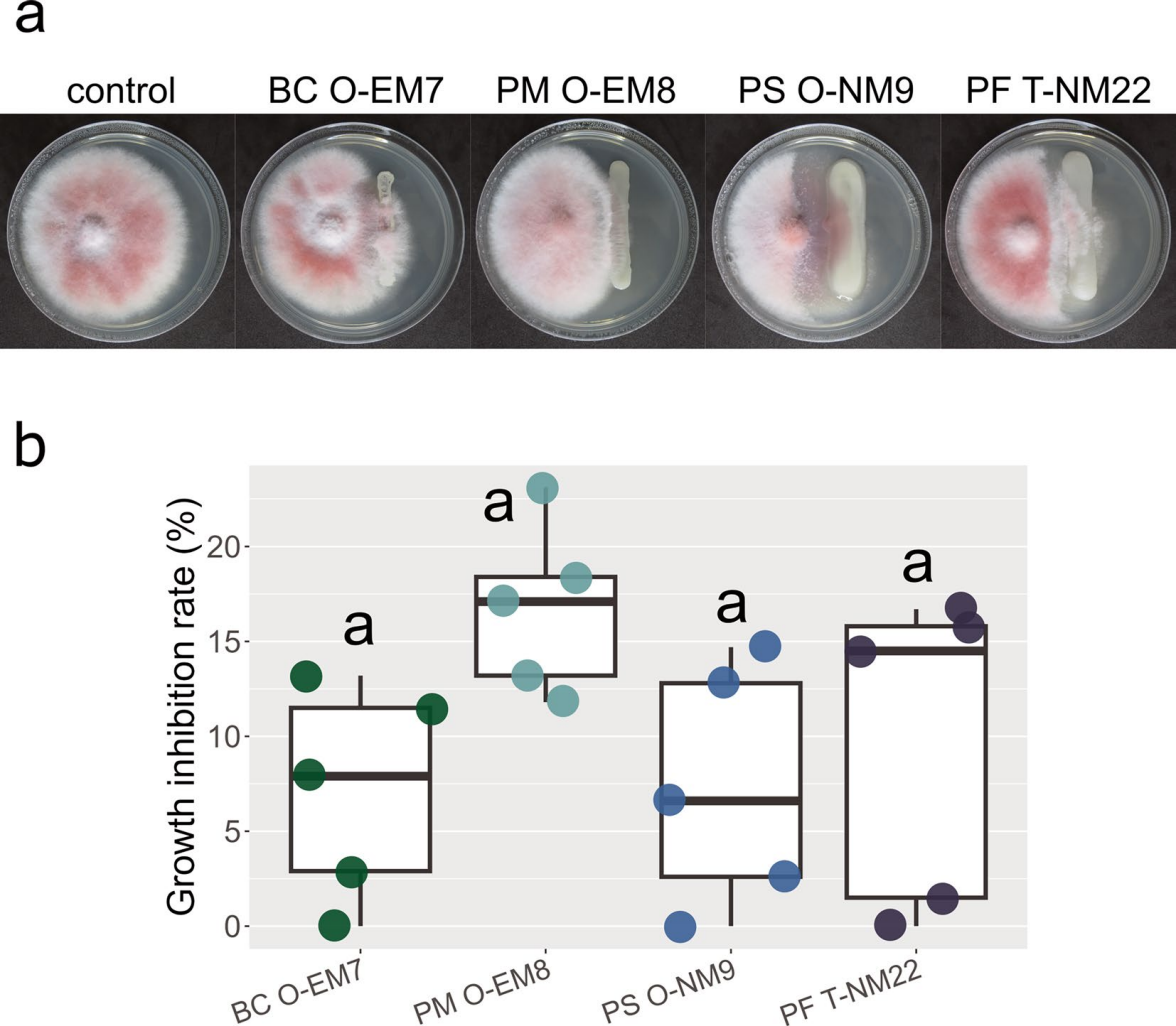
Retarding effect of rhizobacterial strains on the growth of *Fusarium* sp. F-1 (the *Fusarium oxysporum* species complex, MAFF No. 235711). **a** Mycelial growth of *Fusarium* sp. F-1 on potato dextrose agar plates after 7 days of co-cultivation with *Bacillus* sp. strain O-EM7 (BC O-EM7), *Paraburkholderia* sp. strain O-EM8 (PM O-EM8), *Paraburkholderia* sp. strain O-NM9 (PS O-NM9), and *Paraburkholderia* sp. strain T-NM22 (PF T-NM22). **b** Growth inhibition rate of the test fungus in dual culture assay (*n* = 5). The letter (a) indicates no significant difference (*P* > 0.05) among the treatments according to Tukey’s HSD test

In dual culture assay, each bacterial strain hindered the mycelial growth of *Fusarium* sp. F-1 at the 7th day of incubation (Fig. 4a). There were no significant differences among them in terms of growth inhibition rate (determined by measuring the mycelial diameter), although PM O-EM8 tended to show a high inhibition rate (Fig. 4b). However, the mycelium layer in the contact zone with bacterial colonies was markedly thin in the treatments of PS O-NM9 and PF T-NM22. Their retarding effect on the mycelial growth was observed even when the growth inhibition rate was 0% (no difference in mycelial diameter between the control and each dual culture treatment) and was absent or relatively weak in those of BC O-EM7 and PM O-EM8 (Fig. 4a).

### Trapping of rhizobacteria by RETs

**Fig. 5:**
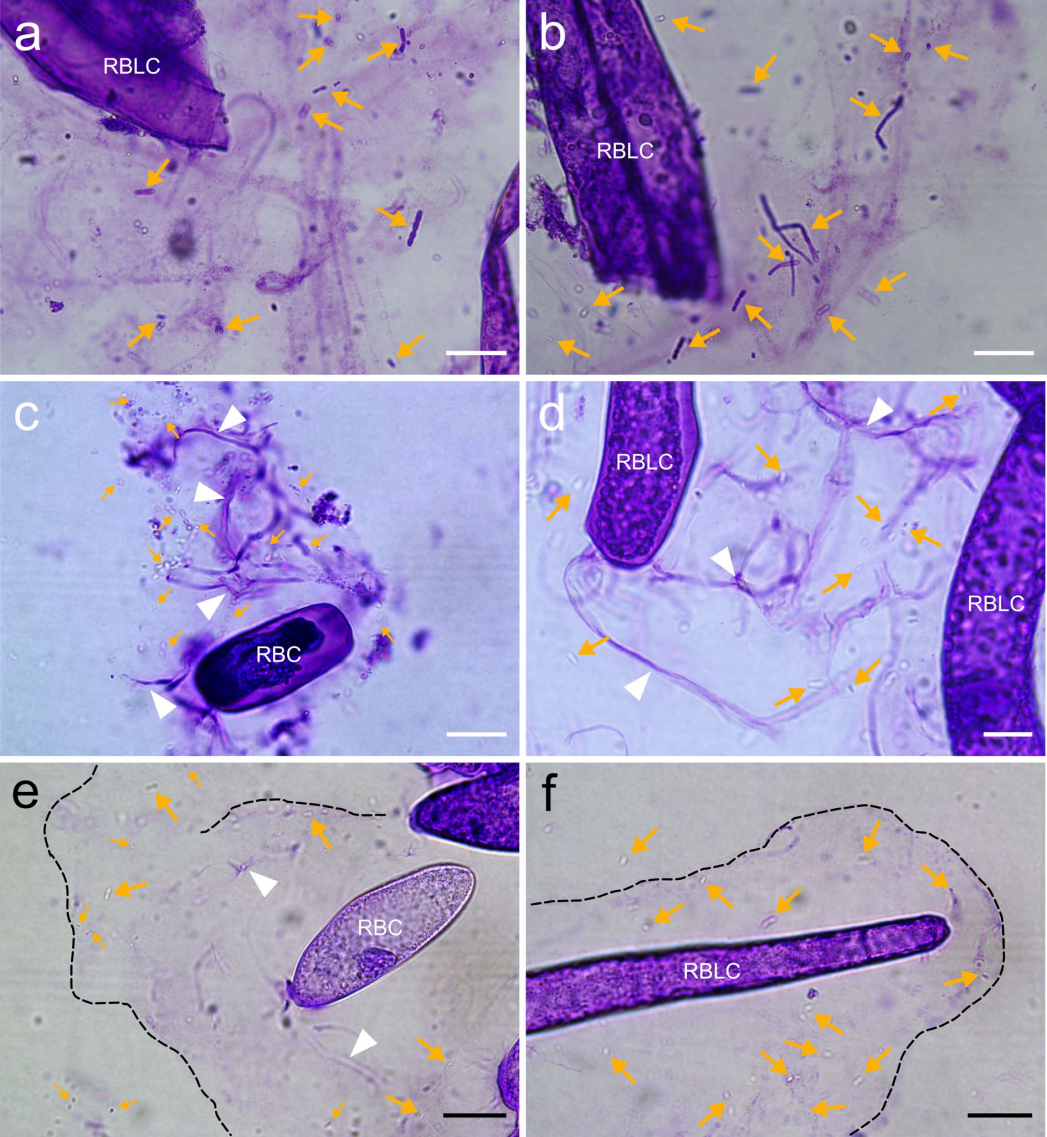
Trapping of rhizobacteria by root mucilage secretions. The primary root and bacterial strains were co-incubated in darkness at 25 ℃ for 2 days. Staining with CV solution revealed the results of co-incubation with *Bacillus* sp. strain O-EM7 (**a**, **b**) and *Paraburkholderia* sp. strain O-NM9 (**c**–**f**). RBC, root border cell; RBLC, root border-like cell. White arrowheads and yellow arrows point to branched strands and bacterial cells, respectively. The black dashed lines in **e**, **f** denote the boundaries of the root mucilage. The images in **c**–**f** were adjusted for brightness and contrast using GIMP. Bars = 20 µm (**a**–**c**, **e**, **f**), 10 µm (**d**)

**Fig. 6:**
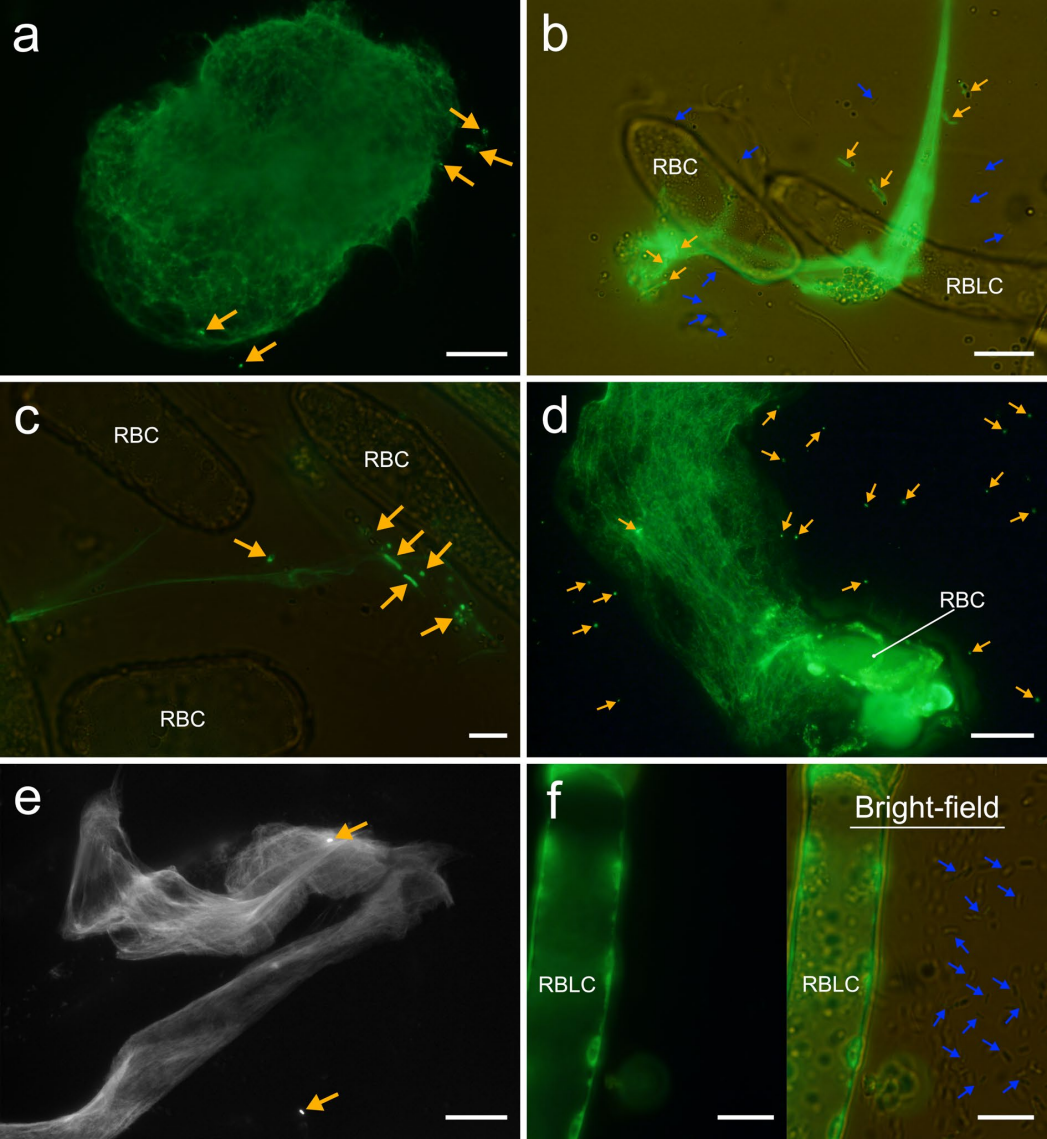
Visualization of the unfolding of extracellular DNA and subsequent trapping of rhizobacteria. The primary root of *Pinus densiflora* and bacterial strains were co-incubated in darkness at 25 ℃ for 2 days. Staining with SYTOX Green revealed the results of co-incubation with *Bacillus* sp. strain O-EM7 (**a**–**c**) and *Paraburkholderia* sp. strain O-NM9 (**d**–**f**). Yellow and blue arrows point to dead and live bacterial cells, respectively. Bars = 50 µm (**d**), 20 µm (**a**, **b**, **e)**, 10 µm (**c**, **f**)

We demonstrated that rhizobacterial cells were trapped by root mucilage and exDNA using CV and SYTOX Green, respectively (Figs. 5, 6). Bacterial cells, which exhibited active swarming in suspension, showed minimal movement after adhering to the mucilage, and were not liberated when the solution was gently agitated (Fig. 5a, b, e, and f; Online Resources S6–S8). Branched strands, similar to frameworks, were also frequently entangled in the mucilage layer with bacterial cells (Fig. 5c, d). SYTOX Green is a DNA-specific dye that cannot penetrate living cell membranes (Wen et al. 2017); hence, only dead cells, including bacteria, show a fluorescence response. SYTOX Green staining visualized some dead bacterial cells trapped by exDNA that had spread in thread-like or web-like structures within the RETs (Fig. 6b–e). However, bacteria trapped by exDNA were localized compared to those in the mucilage layer, and many living bacterial cells attached to the mucilage were observed under bright-field conditions (Fig. 6b, f).

**Fig. 7:**
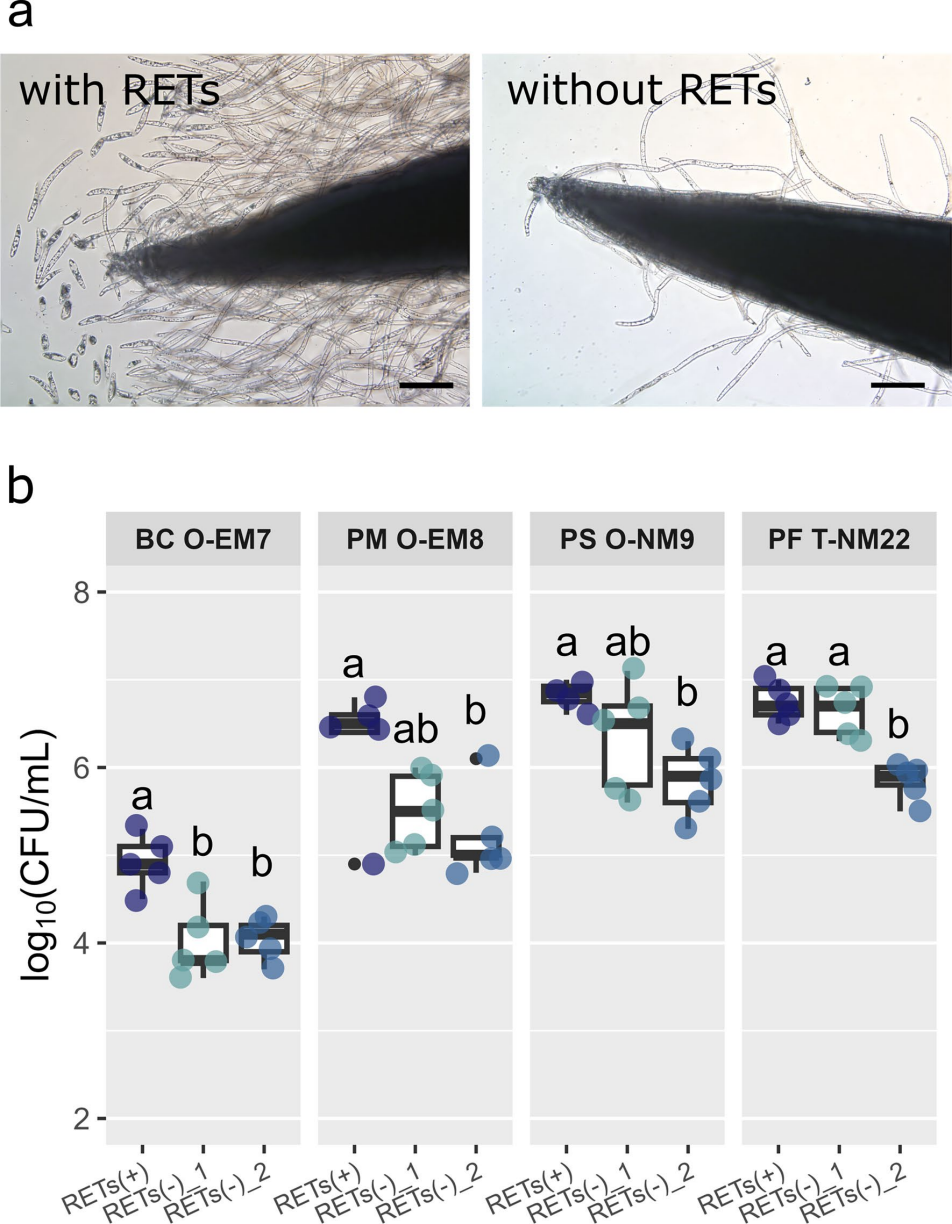
**a** Primary root tip of *Pinus densiflora* before and after the RETs were removed. Bars = 200 µm. **b** Colony forming units in the rhizosphere of *P*. *densiflora* after 2 days of co-incubation with the rhizobacterial strains (*n* = 5) *Bacillus* sp. strain O-EM7 (BC O-EM7), *Paraburkholderia* sp. strain O-EM8 (PM O-EM8), *Paraburkholderia* sp. strain O-NM9 (PS O-NM9), and *Paraburkholderia* sp. strain T-NM22 (PF T-NM22). In **b**, three treatments are compared: RETs (+), control treatment (no RET removal); RETs (−)_1, removal of RETs before the co-incubation; and RETs (−)_2, removal of RETs after the co-incubation. Different letters (a, b) indicate significant (*P* < 0.05) differences among the treatments according to Tukey’s HSD test

It is apparent from Fig. 7a that the removal of RETs reduced the number of AC-DCs and their secretions around the root tip. The CFU assay demonstrated that the presence of RETs significantly increased the CFU counts in the rhizosphere of *P. densiflora* compared to removing them in all bacterial strains (Fig. 7b). With the removal treatment, the CFU counts tended to be lower after versus before incubation, while the suspension of PF T-NM22 differed significantly. Additionally, there were no differences in the morphological features of bacterial colonies formed on the medium among these treatments.

### Production of ROS in response to rhizobacteria

**Fig. 8:**
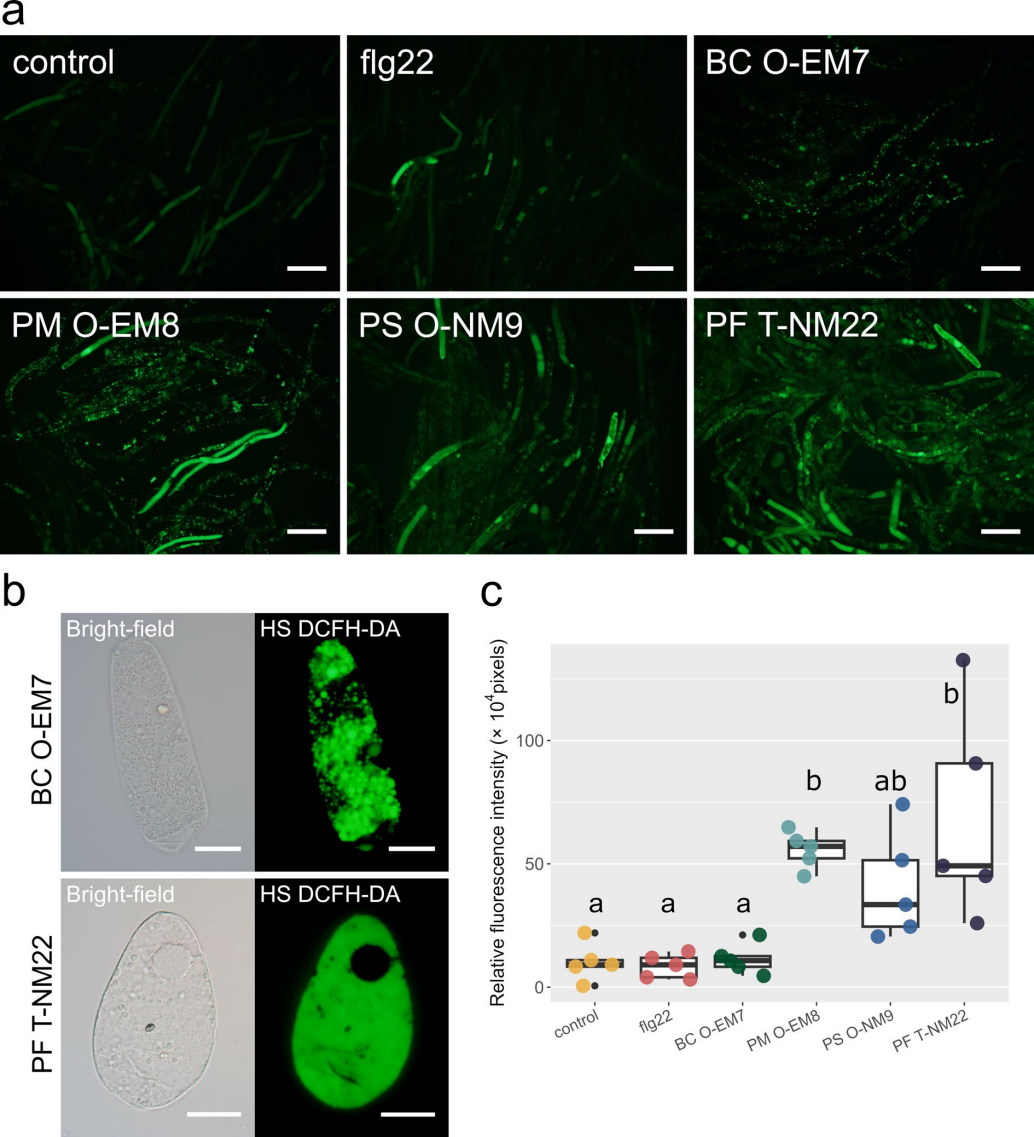
**a** Production of ROS in AC-DCs from *Pinus densiflora* in the early growth stage after various treatments: Control, no treatment; flg22, incubation with the peptide flg22; BC O-EM7, incubation with *Bacillus* sp. strain O-EM7; PM O-EM8, incubation with *Paraburkholderia* sp. strain O-EM8; PS O-NM9, incubation with *Paraburkholderia* sp. strain O-NM9; PF T-NM22, incubation with *Paraburkholderia* sp. strain T-NM22. Bars = 100 µm. **b** Fluorescent patterns of RBCs that showed ROS bursts, detected using the fluorescent probe ROS Assay Kit -Highly Sensitive DCFH-DA- (HS DCFH-DA). Bars = 20 µm. **c** Relative fluorescence intensity of total ROS in the AC-DCs from the early growth stage of *P*. *densiflora* in response to inoculation of each bacterial strain (*n* = 5). Different letters (a, b) indicate significant (*P* < 0.05) differences among the treatments and bacterial strains, according to Tukey’s HSD test

Fluorescent staining for total ROS revealed the production of ROS in AC-DCs (Fig. 8). An early fluorescence response to bacterial MAMP perception, the oxidative burst (Boller and Felix 2009; Zipfel 2009), could be roughly divided into two patterns based on the fluorescence signal: in the first pattern, fluorescence was isolated to cellular organelles; in the second pattern, fluorescence was observed in the whole cell, except the nucleus (Fig. 8b). A comparison of the relative fluorescence intensity based on image analysis revealed that co-incubation with rhizobacterial strains tended to enhance the total ROS production of AC-DCs (Fig. 8c). Two *Paraburkholderia* strains, PM O-EM8 and PF T-NM22, significantly differed from the control treatment, and the latter had the highest values. Strains BC O-EM7 and PS O-NM9 did not result in significant differences in the fluorescence response compared to the control group, and yet these responses were also detected in a wide range of intracellular organelles, similar PM O-EM8 and PF T-NM22 (Fig. 8a, c). In addition, the ROS levels detected after flg22 treatment did not differ significantly from the control and were lower than those after co-incubation with the bacterial suspensions.

## Discussion

The number of AC-DCs produced and the pattern they form vary among plant species. For instance, the root cap of *P*. *sativum* produces many RBCs, whereas *A*. *thaliana* mainly releases cells connected in layers (Hawes et al. 1998; Vicré et al. 2005; Driouich et al. 2019). Hamamoto et al. (2006) demonstrated that these differences among dicotyledonous angiosperms are attributable to variations in RAM organization structures. Meanwhile, Carreras et al. (2020) observed that two Fabaceae tree species, *A*. *raddiana* and *T*. *indica*, have open RAMs, similar to *P*. *sativum*, and yet release mainly sheaths of RBLCs. Our observations of the woody gymnosperm* P*. *densiflora* matched neither *A*. *thaliana* nor *P*. *sativum* but were relatively similar to *A*. *raddiana* among the species studied to date. Therefore, our results partly corroborate those of Hamamoto et al. (2006) in that *P*. *densiflora* has a different RAM structure than *A*. *thaliana* and *P*. *sativum* (Imaichi et al. 2018). These findings suggest that additional factors, such as differences between angiosperms and gymnosperms, differences between dicots and monocots, and the types of symbioses with microorganisms should be considered in addition to the RAM structure. Furthermore, the pectin-degrading enzymes pectin methylesterase and polygalacturonase are reportedly involved in such cell separation (Hawes and Lin 1990; Wen et al. 1999; Driouich et al. 2007). In *A*. *thaliana*, Kamiya et al. (2016) showed that three NAC transcription factors, SOMBRERO, BEARSKIN (BRN) 1, and BRN2, regulate the expression of *ROOT CAP POLYGALACTURONASE* (*RCPG*) in polygalacturonase secretion; of these, at least BRN1 directly binds to the *RCPG* promoter. Karve et al. (2016) also revealed that the transcription factor NIN-LIKE PROTEIN7 controls the cell wall-loosening enzyme *CELLULASE5*, thereby enabling the release of RBLCs in *A*. *thaliana*. Comparing the expression dynamics of target genes among plant species may help categorize their cell detachment patterns.

In *Pinus* species, the viability and long-term survival of AC-DCs still require examination, although such cells have been assessed immediately after detachment (Hawse and Pueppke 1986). Our tests, performed under non-nutritional liquid conditions, support the previous study, which used soybean (*Glycine max* [L.] Merr.) and *P*. *sativum* (until 31 days), and suggest that the AC-DCs of *P*. *densiflora* could survive for more extended periods under favorable conditions. In addition, given that RET components are produced from living AC-DCs (Driouich et al. 2019), these findings indicate that soil nutrient deficiencies or drought stress cause dysfunction of RETs under field conditions and may lead to the death of young seedlings.

We confirmed that the strand structures were not stained with either of two DNA-specific fluorescent probes. A recent study described entangled strands readily stained with CV as “barbed wire” (Wen et al. 2017) structures that could be digested by DNase I or II (Wen et al. 2017; Huskey et al. 2019). Moreover, Ropitaux et al. (2019) reported that cellulose and xyloglucan are present as a dense fibrous network in the root mucilage and maintain AC-DC attachments. These findings are reasonably consistent with our histochemical observations using calcofluor white (CW, staining β-linked polysaccharides) and phalloidin (staining F-actin), indicating that the strands observed in this study differ from exDNA, and their origin is the cell wall or cytoskeleton. Although the role of branched strands in RETs remains unclear, we postulate that they strengthen the structure of mucilage and function as a physical scaffold that binds bacteria, which are found frequently in the mucilage layer. This hypothesis does not conflict with the facts that RBCs can show selectivity for bacteria (Hawes and Pueppke 1989) and root mucilage is a carbon source for rhizobacteria, influencing their community compositions (Knee et al. 2001; Benizri et al. 2007). Thus, these strands may be considered a nonlethal and mucilage-coated component for rhizobacterial trapping, which implies that AC-DCs have some functions even after cell death.

Based on our observations, bacterial cells did not survive trapping by exDNA, while non-trapped ones were alive in the suspensions. These results are consistent with exDNA having contact-mediated antibacterial activity that sequester surface-bound cations and disrupts membrane integrity (Halverson et al. 2015). We also observed that the exDNA derived from the cell nucleus was unfolding to web-like structures, suggesting that it accompanies histone H4 as extracellular chromatin. This protein is the only DNA-binding protein found in plant cells, including AC-DCs, and induces microorganism death by disrupting their cell membranes (Wen et al. 2007; Hawes et al. 2012; Driouich et al. 2019; Monticolo et al. 2020). Thus, exDNA from AC-DCs in *P*. *densiflora* likely has a lethal effect against bacteria. Nevertheless, such bactericidal trappings by exDNA on the whole of the RETs were localized. CFU assay-based results supported these observations. Consequently, the RETs of *P*. *densiflora* should facilitate bacterial colonization in the rhizosphere despite being capable of killing bacteria. Tran et al. (2016) reported that pea and tomato (*Solanum lycopersicum* L.) RBCs released bactericidal exDNA in response to plant pathogenic bacteria, *Ralstonia solanacearum*, while non-pathogenic ones, including *Pseudomonas aureofaciens*, did not trigger their release. Our results accorded with this, in that the selected bacterial strains have no pathogenicity, suggesting that the availability of specific signals that trigger host plant exDNA release and the ability to circumvent them play a vital role in the colonization of the rhizosphere. Therefore, future studies should focus on the immune-evasive and -suppressive capabilities of rhizobacteria, i.e., degrading exDNA, hiding MAMPs, and modulating hormonal signaling pathways, to fully elucidate the effects of exDNA against them (Tran et al. 2016; Yu et al. 2019; Teixeira et al. 2021).

The three *Paraburkholderia* strains retarded the mycelial growth of the plant pathogen *Fusarium* sp. F-1. They also induced ROS production from the host tree AC-DCs of the *P*. *densiflora*. In particular, PF T-NM22 showed key results in both assays. These results support the notion that this bacterial genus primes plant immune responses, functioning as a first line of defense against pathogens (Carrión et al. 2018; Tringe 2019; del Carmen Orozco-Mosqueda et al. 2022; Leitão et al. 2022). Whereas *Bacillus* sp. strain BC O-EM7 also had similar reactions in the assays, these were lower or weaker than those of three *Paraburkholderia*. As shown in Fig. 4a, the three *Paraburkholderia* strains produced large amounts of exopolysaccharides (EPSs) during proliferation. These secretions are produced by various bacteria and secreted into the extracellular environment (Leigh and Coplin 1992). Previous studies revealed that beneficial EPSs play essential roles in the rhizosphere of host plants, functioning as an elicitor, improving biotic and abiotic stress tolerance, and enhancing phytohormone production (Park et al. 2008; Naseem et al. 2018; Bhatia et al. 2021). Thus, our results may partly be explained by differences in production quantity and properties of EPSs among the strains. Additionally, a recent study on *A*. *thaliana* elucidated a feedback loop, that is, an interaction between host plant root and beneficial bacteria; the study showed that bacterial colonization elicited a root immune response and ROS production, followed by auxin stimulation, thereby promoting bacterial survival in the rhizosphere (Tzipilevich et al. 2021). Our results may be relevant to this cycle in that the bacterial strains were not excluded from the rhizosphere, yet further studies are required to reveal whether the loop is also occurring at the RETs.

The present findings reveal that RETs function in the early growth stage of *P*. *densiflora*, and the rhizobacteria trapped by them indirectly contribute to host root defense, including their growth-retarding effect on pathogens. To our knowledge, this is the first study to investigate RETs in an ECM woody gymnosperm and their influence on rhizobacterial colonization, indicating that root-associated bacteria could be a component of RETs, which is equivalent to “an additional layer of the plant immune system” (Teixeira et al. 2019). However, our findings are limited to only the primary roots, given that the root morphology changes anatomically during growth and turnover (Brunner and Scheidegger 1992; McCrady and Comerford 1998; Peterson et al. 1999), and the mechanisms of metabolites vary among root zones within an individual root (Sasse et al. 2018). Thus, it would be beneficial to investigate whether RETs function in mature lateral root systems.

### Supplementary Information

Below is the link to the electronic supplementary material.

Supplementary file1 (PDF 3482 KB)

Supplementary file2 (DOCX 12 KB)

Supplementary file3 (MP4 20578 KB)

Supplementary file4 (MP4 25246 KB)

Supplementary file5 (MP4 13189 KB)

Supplementary file6 (MP4 15487 KB)

Supplementary file7 (MP4 7521 KB)

Supplementary file8 (MP4 13763 KB)

Supplementary file9 (MP4 12050 KB)

Supplementary file10 (MP4 9735 KB)

## Data Availability

The data generated and analyzed in this study are available from the corresponding author upon reasonable request.

## Abbreviations

AC-DCs: Root-associated, cap-derived cells
CFU: Colony-forming unit
CV: Crystal violet
exDNA: Extracellular DNA
RAM: Root apical meristem
RBC: Root border cell
RBLC: Root border-like cell
RETs: Root extracellular traps
ROS: Reactive oxygen species
