# Supplementary material for: Visualization of root extracellular traps in an ectomycorrhizal woody plant (Pinus densiflora) and their interactions with root-associated bacteria
Source: Planta. 2023 Nov 7;258(6):112. doi: 10.1007/s00425-023-04274-1 (PMC10630192; doi:10.1007/s00425-023-04274-1)
Supplement: Supplementary file 1 — Supplementary file1 (PDF 3482 KB) [file 425_2023_4274_MOESM1_ESM.pdf]

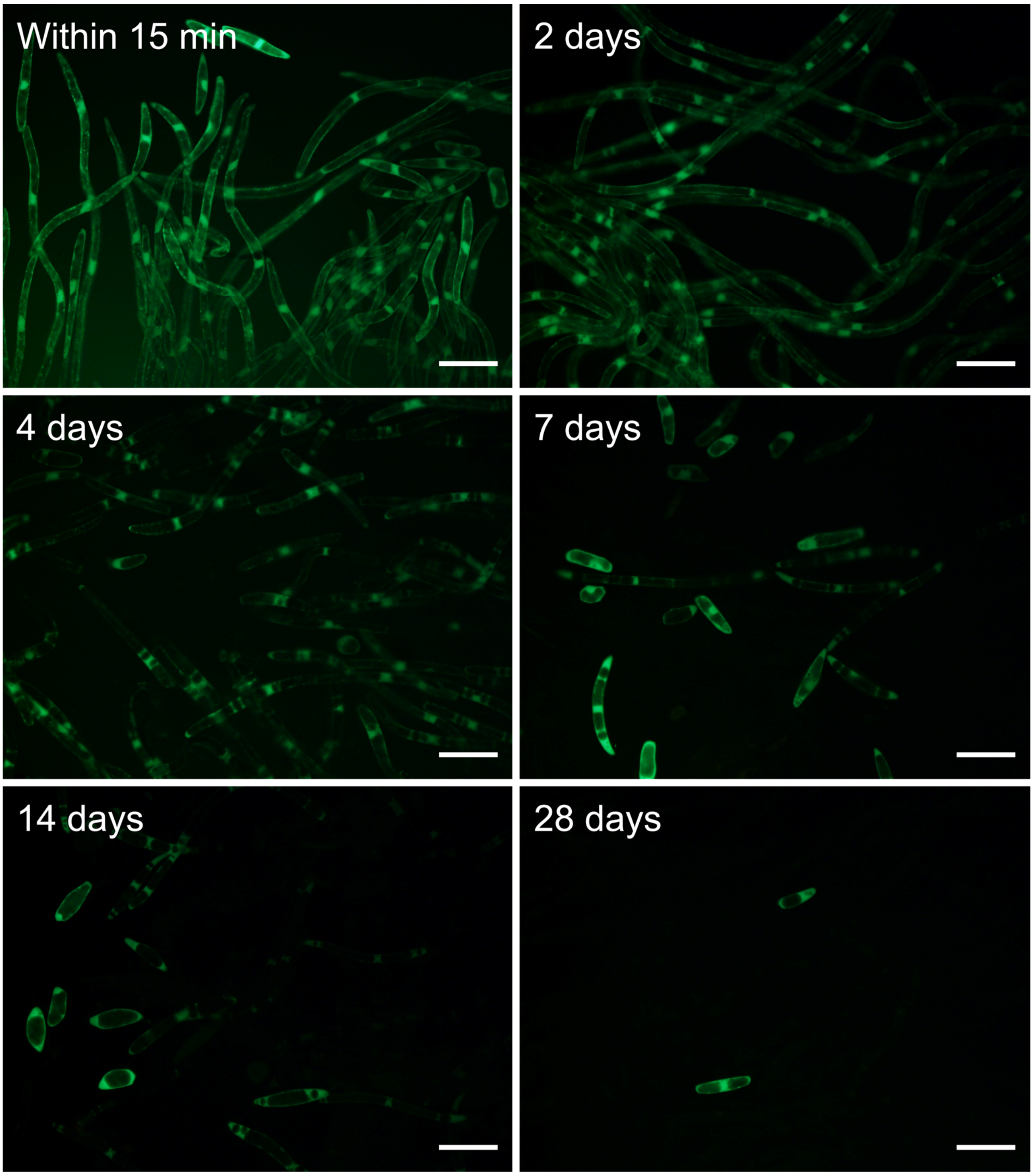

Fig. S1 Cell viability of root-associated, cap-derived cells (AC-DCs) from the early growth stage of *Pinus densiflora*. AC-DCs separated from the root cap were incubated in a 1.5 mL tube containing 200 µL sterile water at 25°C within 15 min and for 2, 4, 7, 14, and 28 days. To identify live versus dead cells, we used fluorescein diacetate solution (1 $\mu g \cdot mL^{-1}$ in phosphate-buffered saline) and 0.01% (v/v) Evans blue solution. Bars = 100 µm

---

Shirakawa M*, Matsushita N, and Fukuda K (2023) Visualization of root extracellular traps in an ectomycorrhizal woody plant (*Pinus densiflora*) and their interactions with root-associated bacteria. *Planta*

* E-mail: mshirakawa941@g.ecc.u-tokyo.ac.jp
