# Supplementary material for: Visualization of root extracellular traps in an ectomycorrhizal woody plant (Pinus densiflora) and their interactions with root-associated bacteria
Source: Planta. 2023 Nov 7;258(6):112. doi: 10.1007/s00425-023-04274-1 (PMC10630192; doi:10.1007/s00425-023-04274-1)
Supplement: Supplementary file 2 — Supplementary file2 (DOCX 12 KB) [file 425_2023_4274_MOESM2_ESM.docx]

**Captions for Online Resources**

**Online Resource S1** Release of root border cells from the root cap and expansion of root border-like cell in response to affusion (sterile water)

**Online Resource S2** Dispersion of root-associated, cap-derived cells and mucilage secretion in India ink solution

**Online Resource S3** Cytoplasmic streaming of root-associated, cap-derived cells immediately after the detachment

**Online Resource S4** Cytoplasmic streaming of root-associated, cap-derived cells after 7 days of incubation (part 1)

**Online Resource S5** Cytoplasmic streaming of root-associated, cap-derived cells after 7 days of incubation (part 2)

**Online Resource S6** Root mucilage encompassing the root-associated, cap-derived cells, with adhered rhizobacterial cells (*Bacillus* sp. strain O-EM7)

**Online Resource S7** Root mucilage encompassing the root-associated, cap-derived cells, with adhered rhizobacterial cells (*Paraburkholderia* sp. strain O-NM9) (part 1)

**Online Resource S8** Root mucilage encompassing the root-associated, cap-derived cells, with adhered rhizobacterial cells (*Paraburkholderia* sp. strain O-NM9) (part 2)
